# Segmentation and Classification of Bone Marrow Cells Images Using Contextual Information for Medical Diagnosis of Acute Leukemias

**DOI:** 10.1371/journal.pone.0130805

**Published:** 2015-06-24

**Authors:** Carolina Reta, Leopoldo Altamirano, Jesus A. Gonzalez, Raquel Diaz-Hernandez, Hayde Peregrina, Ivan Olmos, Jose E. Alonso, Ruben Lobato

**Affiliations:** 1 Department of Computer Science, National Institute for Astrophysics, Optics, and Electronics, Puebla, Mexico; 2 Faculty of Computer Science. Autonomous University of Puebla, Puebla, Mexico; 3 Department of Hematology, Mexican Social Security Institute, Puebla, Mexico; Queen's University Belfast, UNITED KINGDOM

## Abstract

Morphological identification of acute leukemia is a powerful tool used by hematologists to determine the family of such a disease. In some cases, experienced physicians are even able to determine the leukemia subtype of the sample. However, the identification process may have error rates up to 40% (when classifying acute leukemia subtypes) depending on the physician’s experience and the sample quality. This problem raises the need to create automatic tools that provide hematologists with a second opinion during the classification process. Our research presents a contextual analysis methodology for the detection of acute leukemia subtypes from bone marrow cells images. We propose a cells separation algorithm to break up overlapped regions. In this phase, we achieved an average accuracy of 95% in the evaluation of the segmentation process. In a second phase, we extract descriptive features to the nucleus and cytoplasm obtained in the segmentation phase in order to classify leukemia families and subtypes. We finally created a decision algorithm that provides an automatic diagnosis for a patient. In our experiments, we achieved an overall accuracy of 92% in the supervised classification of acute leukemia families, 84% for the lymphoblastic subtypes, and 92% for the myeloblastic subtypes. Finally, we achieved accuracies of 95% in the diagnosis of leukemia families and 90% in the diagnosis of leukemia subtypes.

## Introduction

Leukemia is a cancer that begins in the bone marrow. It is caused by an excessive production of immature leucocytes that replace normal blood cells (leukocytes, red blood cells, and platelets). It causes the body to be exposed to many diseases with no possibility to fight them because of a lack of defenses.

Without treatment, this cancer is the cause of many deaths. In Mexico, according to statistics reported by INEGI [1] in 2006, leukemia was the fifth and sixth cause of death in men (7%) and women (5.8%) with cancer, and it was the first cause of death in children with cancer between 1–4 and 5–14 years old, with 48.5% and 52.2% of deceases, respectively.

In the diagnosis of leukemia, in addition to consider the signs and clinical symptoms of the patient, it is necessary to perform a clinical test to detect the presence of abnormal cells. A blood count study of peripheral blood samples allows obtaining the amount and percentages of different types of blood cells (red cells, white cells, and platelets). If there are abnormalities in this count, a morphological bone marrow smear analysis is done to confirm the presence of immature leukemic cells. In this study, a pathologist observes some cells samples under light microscopy looking for abnormalities presented in the white blood cells in order to detect the existence of leukemia and predict its type and possible subtype. This classification is very important as it determines the treatment prescribed to the patient. This study may have an error rate between 30% and 40% depending on the pathologist experience and the difficulty to distinguish leukemia types and subtypes [2–3]. Despite flow cytometry is one of the most reliable techniques to establish accurate diagnoses of acute leukemia subtypes, still in many hospitals of the third world countries this type of study is not available, mainly those hospitals which belong to the public sector [4]. In hospitals where they have the equipment, because of the high percentage of studies to be performed daily, very often only those samples from patients where microscopic analysis has determined the possible existence of disease are analyzed to determine the type of leukemia that the patient presents and confirm the diagnosis. On the other side, a lot of acute cases of the disease are detected in low-income people, which have limited access to private hospitals, allowing the progress of the disease to later stages due to the absence of opportune diagnosis. The purpose of this work, besides being a reinforcement of a reliable diagnosis of this type of cancer, is to provide a method for detecting the disease fast and with high accuracy, providing to the population in general an inexpensive alternative to obtain an opportune diagnosis and treatment.

The four main types of leukemia are: acute lymphocytic leukemia (ALL), acute myeloblastic leukemia (AML), chronic lymphocytic leukemia (CLL), and chronic myeloblastic leukemia (CML). Each main type of leukemia is named according to the type of cell that is affected (a lymphoid cell or a myeloid cell) and whether the disease begins with a mature or immature cell. Acute leukemias are fast-growing and can overrun the body within a few weeks or months. By contrast, chronic leukemias are slow-growing and progressively worsen over the years.

Early detection of acute leukemia allows the physician to prescribe an appropriate treatment to the patient. This is decisive due to the quick development of the disease. According to the FAB classification [5], there are 11 subtypes of acute leukemia. This morphological classification includes 3 subtypes for ALL (L1, L2, and L3) and 8 subtypes of AML (M0, M1, M2, M3, M4, M5, M6, and M7).

In this work, we are especially interested in automatically determining the type and subtype of acute leukemias by analyzing information contained in digital images of bone marrow smears. Some important tasks to be undertaken in this work are: 1) the extraction of the nucleus and cytoplasm of cells from blood smear images showing heterogeneous staining; 2) the detection of overlapped cells in images with high-cell population; 3) the definition of morphological, statistical, texture, and size ratio features of the nucleus and cytoplasm that describe the different blood cells; 4) the generation of classification models to recognize acute leukemia cells through the learning of their descriptive features; and 5) the model of a decision algorithm for the diagnosis of types and subtypes of acute leukemia based on the results of the generated classifiers.

It is noteworthy that this paper does not cover all of the acute leukemia pathologies, but those directly related to images samples provided by [6], corresponding to 5 different subtypes of acute leukemia. The results of this work will show the importance of using contextual information in the analysis of real images using computer vision, data mining, and data fusion techniques. Likewise, these results may be applied in the medical sector, specifically in the hematologic area to detect subtypes of acute leukemia.

## Related Work

Segmenting the nucleus and cytoplasm of leukocytes from bone marrow images is a very difficult task, as the images show heterogeneous staining and high-cell population. Some segmentation techniques such as thresholding, edge detection, pixel clustering, and regions growing have been combined to extract the nucleus and cytoplasm of leukocytes [7–11]. These techniques could be applied as the images showed uniform backgrounds and high contrast that appropriately defined the objects of interest. However, it is common that samples generated for daily studies in hematology laboratories are not uniform (non-uniform staining, different color for cells and backgrounds, and others problems). Because of this, in our work there are images with low contrast among cell elements and a variety of colors and textures that make cellular elements difficult to distinguish. For this reason, we propose a segmentation algorithm based on color and texture pixels features that can work in bone marrow images showing heterogeneous staining. Some works combine techniques such as SVM, mean-shift, and color image segmentation, with the goal of obtaining an automatic machine-learning method to find models of the different parts of the image (i.e. nuclei, mature erythrocytes, and background). These methods achieve higher segmentation accuracy in complex scenes and are more robust to color confusion and changes compared with the thresholding and the watershed algorithms [12]. More recently, a variation of this method using simulated visual attention via learning by on-line sampling was proposed by [13].

Regarding the problem of overlapped blood cells, few algorithms have been proposed. These algorithms split cells either by joining concave points using separating lines [8, 14] or by eroding and growing regions retaining the shape [11, 15]. In this paper we also propose a cell separation algorithm that keeps the original shape of the blood cell and uses information of this shape to split the overlapped regions by drawing a conical curve.

Few solutions have been proposed to the classification of leukemia cells. Morales [2] and Galindo [4] extracted geometric, statistical, and texture features from whole cells and classified them into types and subtypes of acute leukemia, respectively, obtaining promising results. Mohapatra et al. [16] proposed the use of an ensemble of classifiers to improve the accuracy in the classification of leukemia cells with the aim of proposing a quantitative microscopic approach to discriminate lymphoblast (malignant) from lymphocytes (normal). On the other hand, in the case of AML, Lim et al. [17] proposed a method that consists of gradient magnitude, thresholding, morphological operations, and watershed transform to perform cells segmentation for leukemia subtypes M2, M5, and M6.

In this paper we propose a leukemia cells classification method by means of a more detailed description of the cells. This requires processing the image pixels to segment the cells and separate their respective nucleus and cytoplasm in order to extract from them features that help to improve the classification among leukemia subtypes.

Segmentation is an important and challenging task in the automatic image analysis of blood cells. This happens because its accuracy has a high impact on the automatic identification and classification of pathologies. As we could not find a good cell segmentation method for bone marrow images, this work proposes a robust solution to the problem. In our solution, we propose a segmentation algorithm that uses contextual information of images pixels to extract the nucleus and cytoplasm of the leukemia cells in images with color and texture variations and high-cell population. By extracting and analyzing features of the nucleus and cytoplasm of cells, we expect to improve the results achieved in previous work. This work also proposes a decision algorithm that uses the contextual information from the images samples of a patient for an automatic diagnosis of type and subtype of acute leukemia.

## Method

Our method analyzes leukemia cells digital images from a contextual approach in order to classify and diagnose five subtypes of acute leukemia. This approach allows us interpreting the visual information of the cellular elements in a similar way to the one that we use as humans to identify objects. The proposed solution for cell morphology analysis follows a methodology that uses computer vision and data mining techniques. This methodology includes the segmentation and identification of cellular elements, and the classification and diagnosis of types and subtypes of acute leukemia.

### Segmentation of cellular elements

The segmentation algorithm aims to separate every blood cell in its two most important elements: nucleus and cytoplasm. Because the color intensity of a pixel is not enough to successfully segment cells from images with color variations, this algorithm uses features of the neighboring pixels as contextual information to generate homogeneous regions.

**Fig 1:**
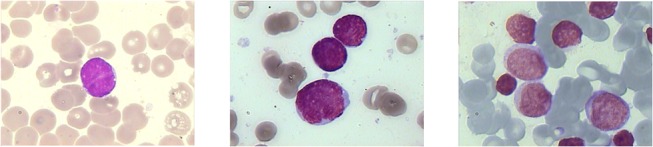
Examples of images with a Wright stain preparation, but with color differences in cells and backgrounds.

With the help of an expert, we analyzed the features of samples of bone marrow cell images with different staining in order to design an algorithm that can appropriately segment leukocytes and their respective nucleus. Our leukemia digital images were obtained from bone marrow samples using the Wright’s stain method. In this analysis we observed some color and texture features that we used to distinguish between blood cells such as: 1) red blood cells get orange and rose shades while leukocytes exhibit purple tonalities in their nucleus, and blue and rose tones in their cytoplasm (for lymphocytes and myelocytes, respectively), 2) the color intensities acquired by the nucleus are darker than those of the cytoplasm, and 3) the texture of the nucleus and cytoplasm of leukocytes, red blood cells, and the image background is different among them (see Fig 1).

#### Color features

**Fig 2:**
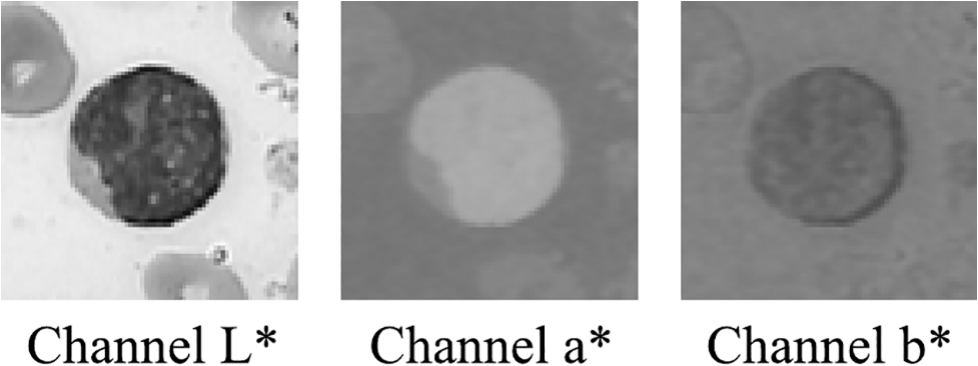
Blood smear in the CIE L*a*b* color space.

According to the previous description, we decided to use the CIE L*a*b* color space. We took this decision because it highlights the visual differences among colors. It also provides a perceptual approach with high accuracy in the color difference calculation process. Since our image collection is in the RGB color space, a transformation from RGB to the CIE L*a*b* space was done using the formulas presented in [18]. Fig 2 shows the CIE L*a*b* color channels for a bone marrow cell image.

As we can see in Fig 2, the luminosity channel L* provides a suitable nucleus color representation that allows identifying objects with different light reflection. We can also note that channel b* provides a suitable color representation for cells in such a way that it highlights elements with purple and blue tonalities.

**Fig 3:**
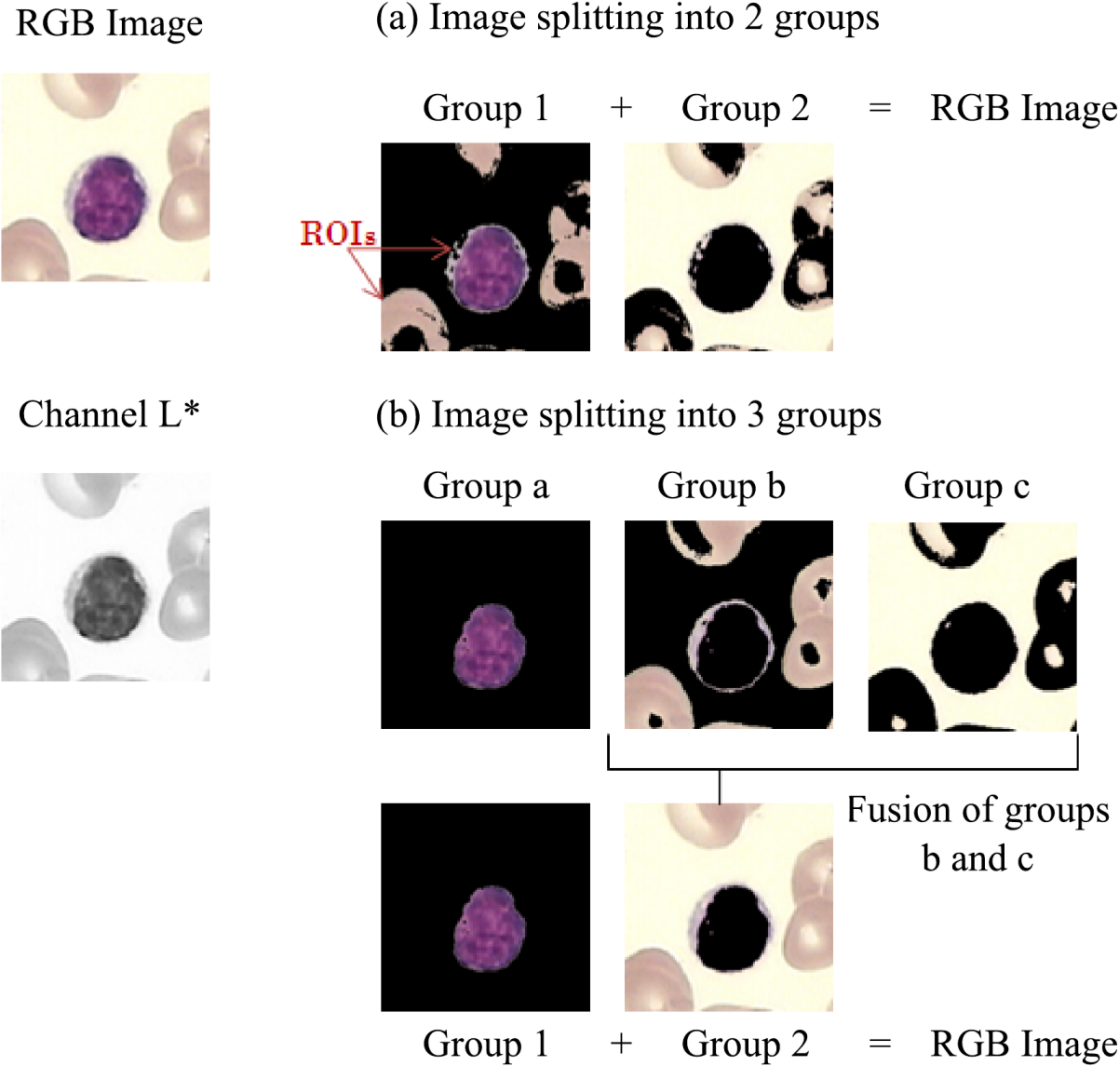
Groups creation using similarity features from a channel intensity.

Since channels L* and b* contain valuable information about the nucleus and the whole cells respectively, they were used to create two similar groups in their channel intensity values using the k-means clustering algorithm with k = 2 and k = 3 (see Fig 3). The first group was established by selecting the cluster that better represents the nucleus or cell information. All the pixels that do not correspond to the first group fall in the second one. We finally calculated the channel intensity statistics-mean, variance, and standard deviation- to each group in order to incorporate them as color features for the segmentation model.

### Texture features

We performed a texture analysis using the Wold’s decomposition model [19] to separate texture into its structural and stochastic components. We chose this model because blood cells images present heterogeneous textures. Hence, both periodical and random textures can be found in such images. Additional motivations for choosing this model were its similarity relation with the human visual perception system, and its invariant properties to translation, rotation, and scale.

**Fig 4:**
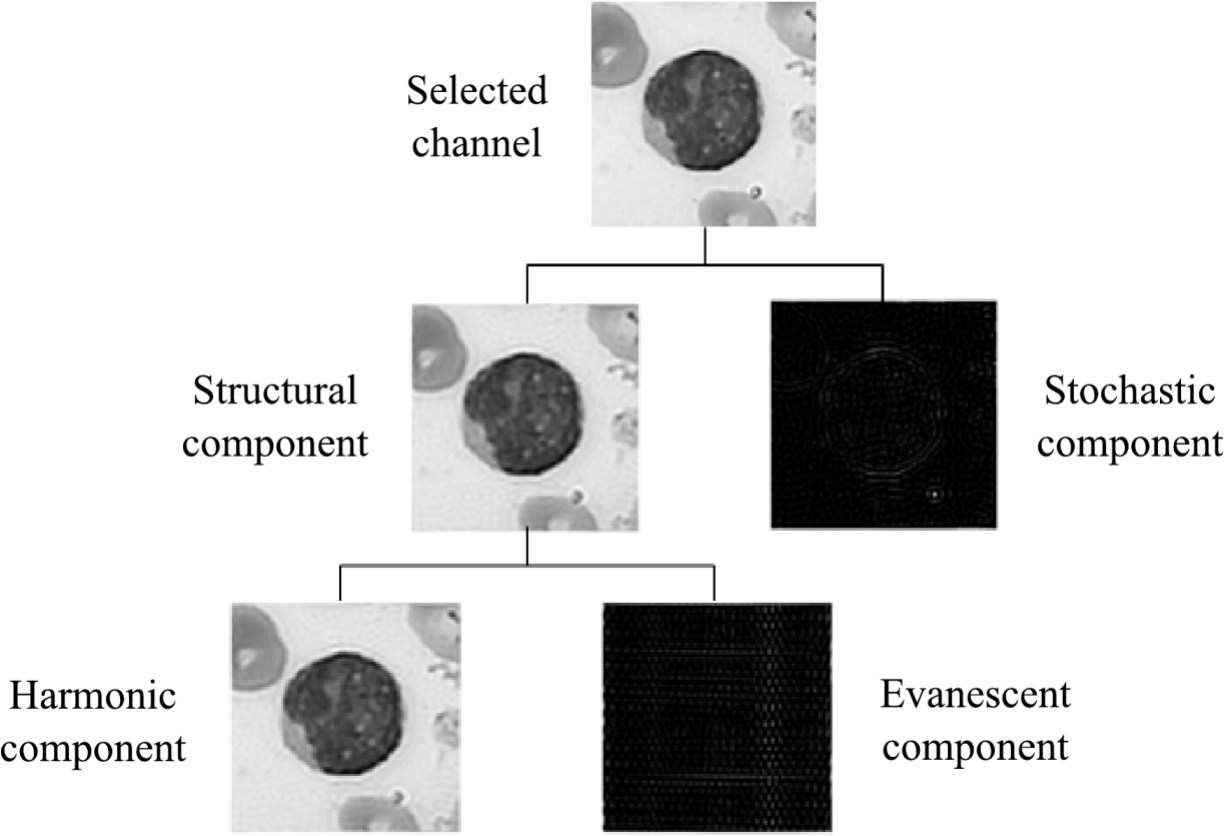
Wold's decomposition texture model.

The Wold’s decomposition model interprets the image texture by means of the sum of three mutually orthogonal components: a harmonic field, a generalized evanescent field, and a stochastic field [19]. The perceptual characteristics of these fields can be described as: periodicity, directionality, and randomness, respectively, according to the three most important human perception dimensions identified by [20]. Fig 4 shows a diagram of the orthogonal components of the Wold’s decomposition model for a selected channel.

**Fig 5:**
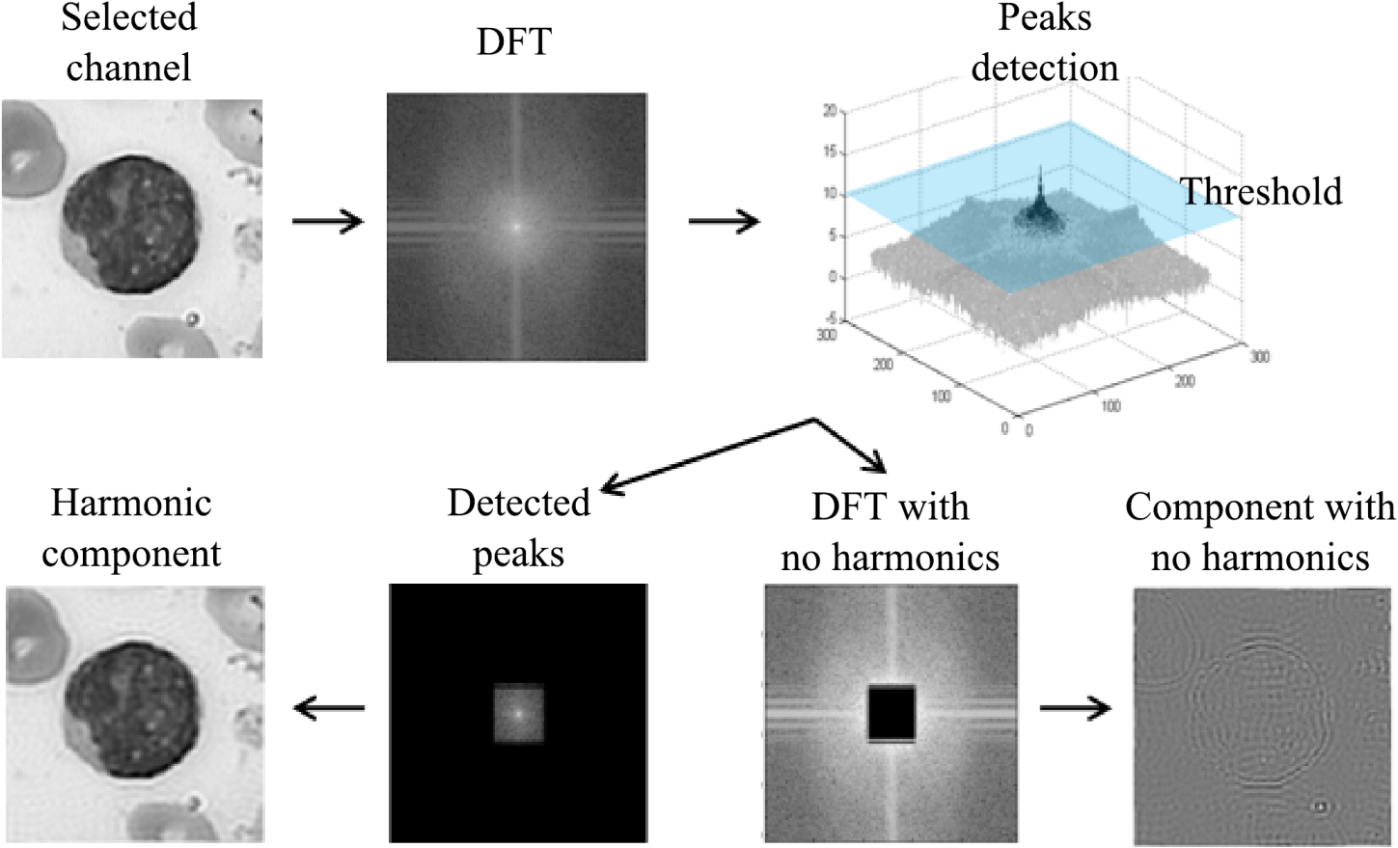
Harmonic field parameterization.

In order to parameterize the harmonic field, we used the method proposed by [19]. In this algorithm (see Fig 5), we first solved the sinusoidal using the discrete Fourier transform (DFT). Next, we located harmonic peaks by identifying the largest isolated peaks in the harmonic frequencies. We established a value of 10 as the amplitude threshold (experimentally), which is sufficient to find the peaks that were considered harmonic components in the cell images. Finally, the harmonic field parameterization was done by evaluating the amplitude and phase values of the DFT from the frequencies identified as peaks.

**Fig 6:**
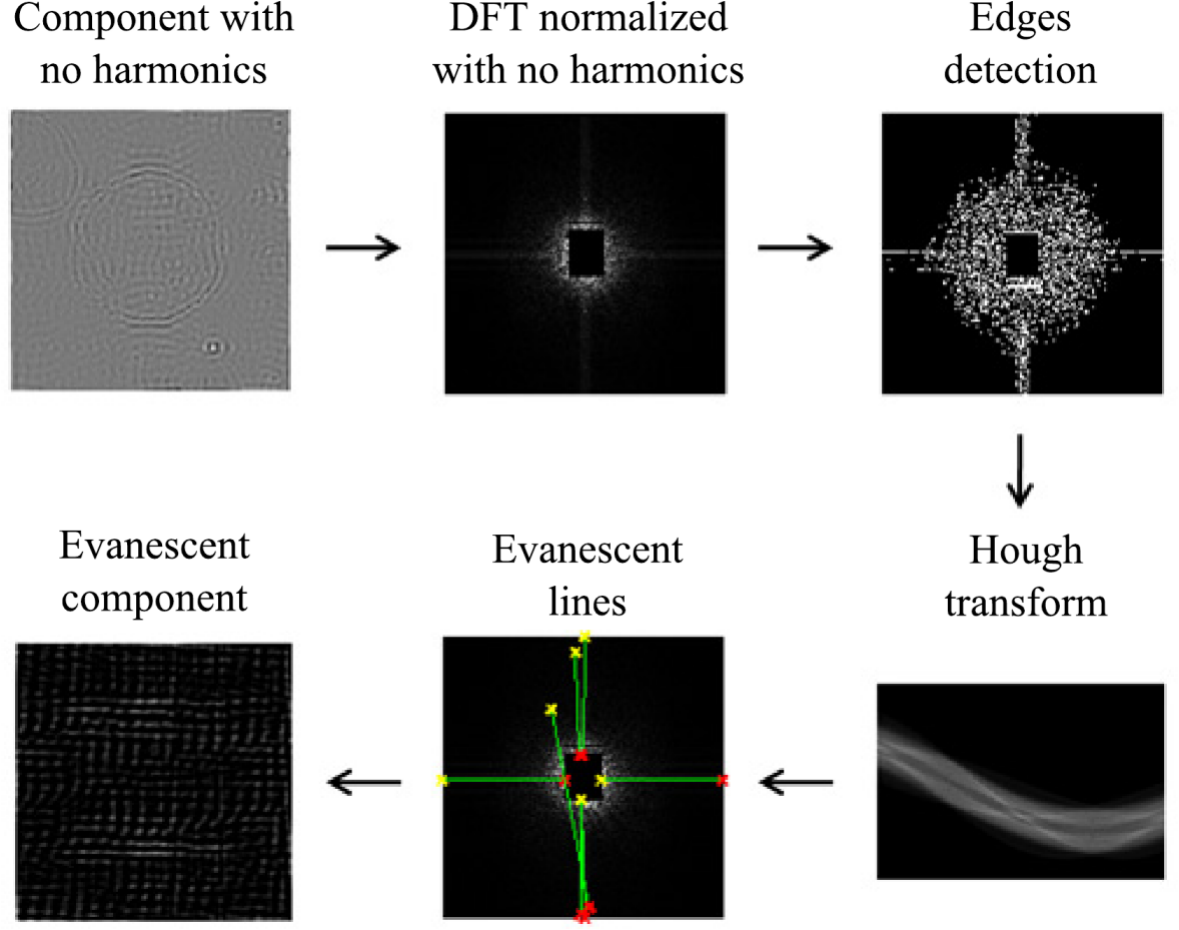
Generalized evanescent field parameterization.

In the parameterization of the generalized evanescent field we used the algorithm proposed by [21]. In this algorithm (see Fig 6), the DFT without harmonic components is used to find four evanescent lines using a Hough transform. The parameterization is carried out by evaluating the amplitude and phase values of the DFT from the frequencies of the evanescent lines identified.

The texture structural component is the sum of the harmonic and generalized evanescent fields. The stochastic component parameterization is done by evaluating the amplitude and phase values of the residual DFT once the structural component is removed.

#### Color and texture segmentation

We propose a segmentation method that uses contextual color and texture information to classify pixels corresponding to cell elements in bone marrow images with heterogeneous staining. For this, we first represent a channel using a binary Markov Random Field model which consists of a label field and three observation fields (channel intensity, structural texture, and stochastic texture fields). After that, according to the Bayes theorem, we represented the segmentation problem as a Maximum a Posteriori (MAP) estimation of the label field. Theoretical details can be found in [22].

**Fig 7:**
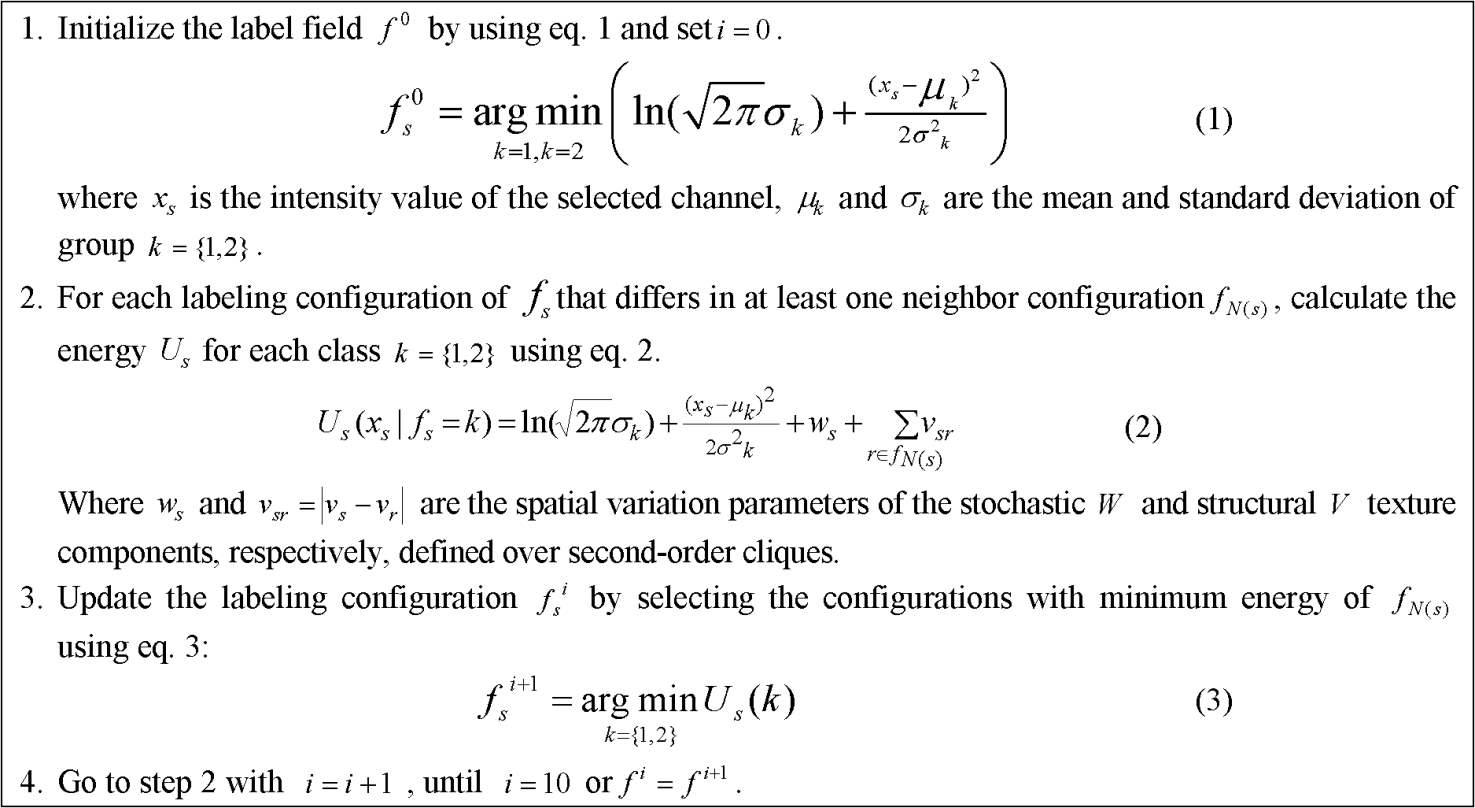
Procedure of MAP estimation for cell segmentation.

Based on the Iterated Conditional Model algorithm [23], we solved the MAP estimation using the procedure shown in Fig 7.

### Cells identification

Contextual information that relates an object with other objects can provide relevant information to the object recognition. This is better than the intrinsic features of the object by itself. This paper proposes to identify the cells by exploiting contextual relationships (spatial and geometric) of the objects in the image. Contextual information such as position, color and shape of the regions can be used to identify the cells. This information is useful because it highlights regularities of the regions allowing the identification of nuclei and cells, and overlapped regions.

The goal of this step is identifying leukocytes through the recognition of their nucleus and cytoplasm. From the regions obtained in the segmentation process, we analyzed their shape, color, and spatial relation with respect to other regions to determine whether an analyzed region is a nucleus or a cytoplasm.

**Fig 8:**
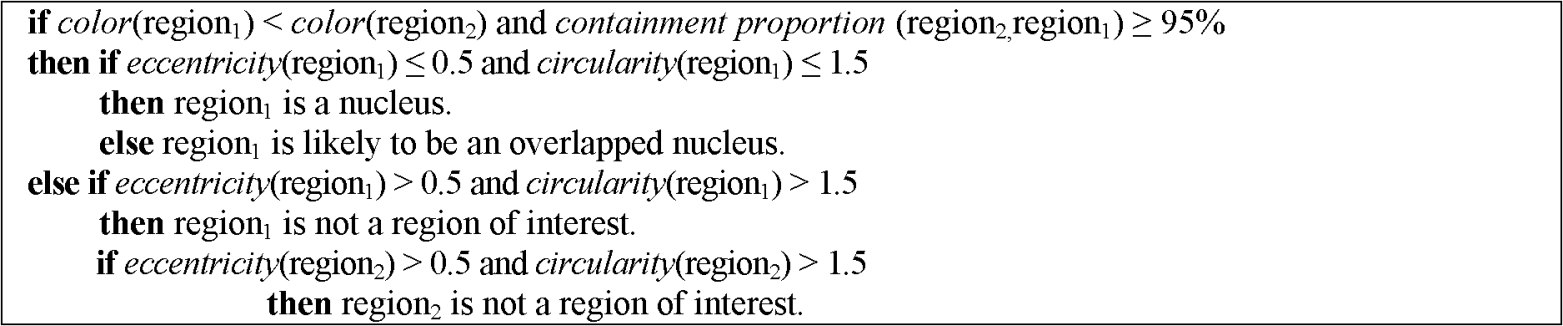
Decision rules to identify nucleus.

**Fig 9:**
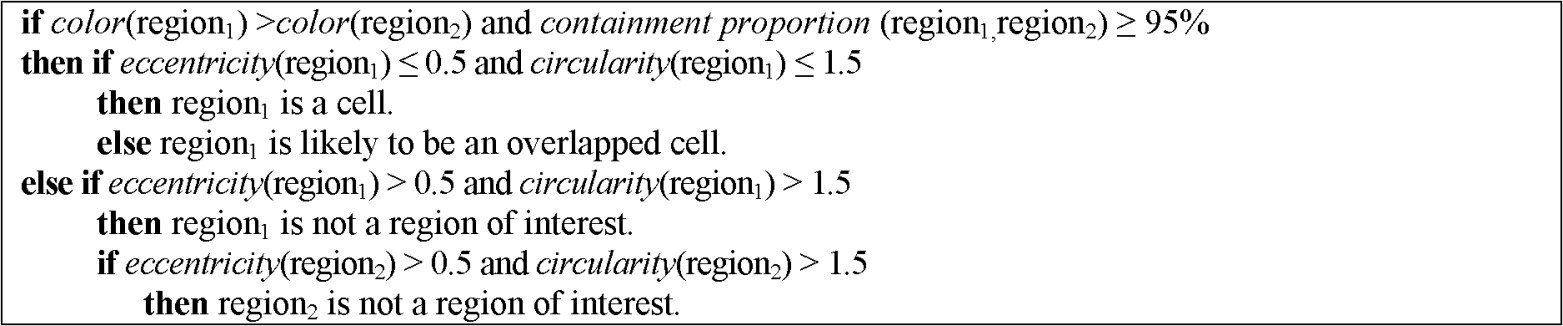
Decision rules to identify cells.

The features that were used to recognize cellular elements are: *circularity* to measure the perimeter complexity of a circular object (circularity = perimeter^2^ / (4πarea)), *eccentricity* to find out how much the object deviates from being circular (eccentricity = dist(center, focus)), *color* to determine if a region is darker than another one, and *containment proportion* to establish whether a region contains or is contained by another region. Using these features and a priori knowledge about the cellular elements structure we designed the rule-based classifiers presented in Figs 8 and 9 to label nucleus and cells. We selected a subset of 20 regions with regular shapes (nucleus and cells) and 20 regions with irregular shapes (overlapped regions) and we generated classification rules (using Weka) that discriminate between these types of forms. These rules gave us an idea of the threshold values to use to determine if a region is likely to be a cellular element. Then, we added the color relation and containment proportion rules so that we could match the cells with their respective nucleus. We established a containment proportion threshold of 95% because some pixels are missed either in the cell segmentation or in the separation of overlapped regions steps.

### Separation of overlapped blood cells

**Fig 10:**
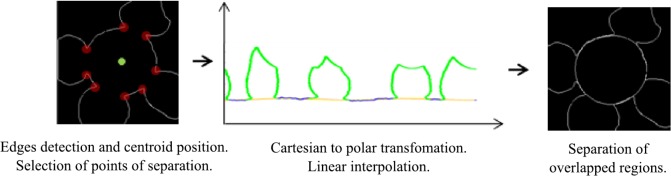
Cell separation procedure.

Fig 10 summarizes the process of cell separation once an overlapped region is identified. In order to split the overlapped regions, we obtain the edges of the region and its centroid to then provide some concave points as points of separation. Then, we transform edges from a cartesian to a polar space, and we interpolate discontinuous points using a linear interpolation. This allows completing cell borders with a conical shape once we come back to the cartesian space. Finally, we join some edges discontinuities by applying morphological operations.

### Classification of acute leukemia cells

The classification of types and subtypes of acute leukemia cells will be done by using several features extracted from regions of cells and their respective nucleus and cytoplasm.

The suitable recognition of leukemia cells requires the definition of good descriptive features that facilitate their classification. In this phase we extract geometric, statistical, texture, and size ratio features from regions obtained in the segmentation process (nucleus, cytoplasm, and whole cell) and we analyze these features to identify the family and subtypes of acute leukemia. It is important to mention that we do not normalize the images to extract these features, since the size and color of the cells are important characteristics to distinguish among subtypes of leukemia. Table 1 shows these features. All the morphological features mentioned in Table 1 were extracted from each nucleus and cell. Since many of the cytoplasm features are included in the cell, we only calculated its area. Due to the morphological analysis performed by the expert, these features are not relevant to the classification of cells. In case of the nucleus, cytoplasm, and cells, we extracted statistical and texture features from the channels of the RGB image and the gray scale image. We obtained another set of features applying principal components analysis to the images corresponding to the nucleus and the cell. We used as features the firsts 10 eigen values for the channels of the RGB image and gray image, which represent at least the 80% of the data variability in each region. We also obtain size ratio features from the cellular elements.

**Table 1:** Representative features for the cell description.

No.	Feature	Type	Description
1	Area	Morphologic	Actual number of pixels in the ROI.
2	Perimeter	Morphologic	Distance between each adjoining pair of pixels around the border of the ROI.
3	Circularity	Morphologic	Complexity of the perimeter of a circular object. Perimeter^2^ / (4*Area*pi).
4	Width	Morphologic	Width of the smallest rectangle containing the ROI.
5	Length	Morphologic	Length of the smallest rectangle containing the ROI.
6	Elongation	Morphologic	Growth in one direction of the ROI. Length / Width.
7	Major axis length	Morphologic	Major axis of the ellipse containing the ROI.
8	Minor axis length	Morphologic	Minor axis of the ellipse containing the ROI.
9	Eccentricity	Morphologic	Ratio of the distance between the foci of the ellipse containing the ROI and its major axis length.
10	Extent	Morphologic	Proportion of pixels in the smallest rectangle that are also in the ROI. Area / (Width*Length).
11	Equivalent diameter	Morphologic	Diameter of the circle with the same area as the ROI.
12	Euler number	Morphologic	Number of objects in the region minus the number of holes in those objects.
13	Convex area	Morphologic	Area of the smallest convex polygon containing the ROI.
14	Solidity	Morphologic	Proportion of pixels in the convex hull that are also in the ROI. Area / Convex area.
15	Mode	Statistical	Most frequent value of the pixels intensity of the ROI.
16	Mean	Statistical	Average value of the pixels intensity of the ROI.
17	Standard deviation	Statistical	Standard deviation of the pixels intensity of the ROI.
18	Variance	Statistical	Variance value of the pixels intensity of the ROI.
19	Sum	Statistical	Sum of the pixels intensity of the ROI.
20	Homogeneity	Texture	Closeness of the distribution of elements in the gray-level co-occurrence matrix to its diagonal.
21	Contrast	Texture	Intensity contrast between a pixel and its neighbor over the whole image.
22	Correlation	Texture	Value that measures how correlated a pixel is to its neighbor over the whole image.
23	Energy	Texture	Sum of squared elements in the gray-level co-occurrence matrix.
24	Entropy	Texture	Value that measures the smoothness of the image in terms of gray-levels.
25–34	Eigen R	Eigen	Firsts 10 eigen values of the R channel of the RGB image.
35–44	Eigen G	Eigen	Firsts 10 eigen values of the G channel of the RGB image.
45–54	Eigen B	Eigen	Firsts 10 eigen values of the B channel of the RGB image.
55–64	Eigen gray	Eigen	Firsts 10 eigen values of the gray image.
65	N-Cyto area	Size ratio	Ratio of the area between the nucleus and the cytoplasm. Nucleus area / Cytoplasm area.
66	N-Cell area	Size ratio	Proportion of nucleus’ area in the cell’s area. Nucleus area / Cell area.
67	N-Cell perimeter	Size ratio	Ratio of the perimeter between the nucleus and the cell. Nucleus perimeter / Cell perimeter.

**Fig 11:**
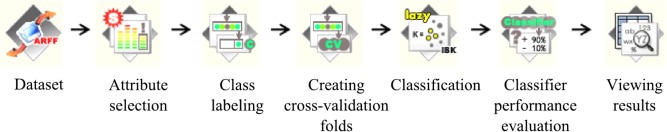
Classification process for acute leukemia subtypes.

The analysis of these features to classify acute leukemia cells was done using different training and testing sets, attributes selection, and classification algorithms available in Weka [24]. We show the classification process in Fig 11. The test sets are described in the experimental results section.

### Diagnosis of acute leukemia

In order to make an acute leukemia diagnosis, physicians previously analyzed different affected leukemia cells through a light microscopy study. Similar to the analysis made by experts to diagnose acute leukemia subtypes, this paper proposes a decision algorithm that combines the results of the classification of acute leukemia cell samples belonging to a patient.

The proposed diagnosis algorithm is not intended to replace the clinical diagnosis performed by the expert, but rather provide him with a second opinion. The expert must also take into account other factors such as the patient's medical history, symptoms, clinical signs, and the results of the morphological study to diagnose acute leukemia according to his experience and to offer an appropriate treatment.

**Fig 12:**
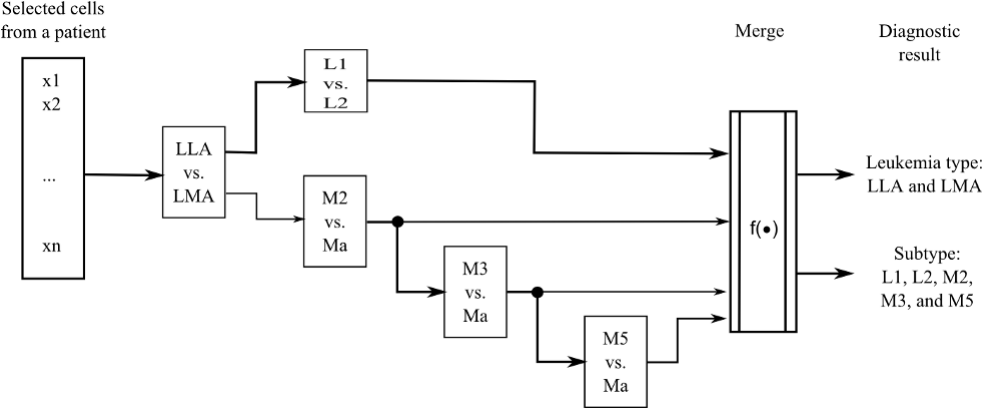
Cascade classification model for the diagnosis of acute leukemia.

Fig 12 shows a simple design for our acute leukemia diagnosis algorithm based on a combination of binary classifiers. In this scheme, the family classification of acute leukemia is first done followed by the subtype classification. We use a majority vote criterion on the predictions of binary classifiers in all the cells samples of the patient to determine the family and subtype of acute leukemia in a patient.

A drawback of the design of Fig 12 is that the decisions made by subtype’s classifiers are dependent on the prediction of the family’s classifiers. So, if the family’s classifier predicts incorrectly, the subtype’s classification will also be incorrect.

**Fig 13:**
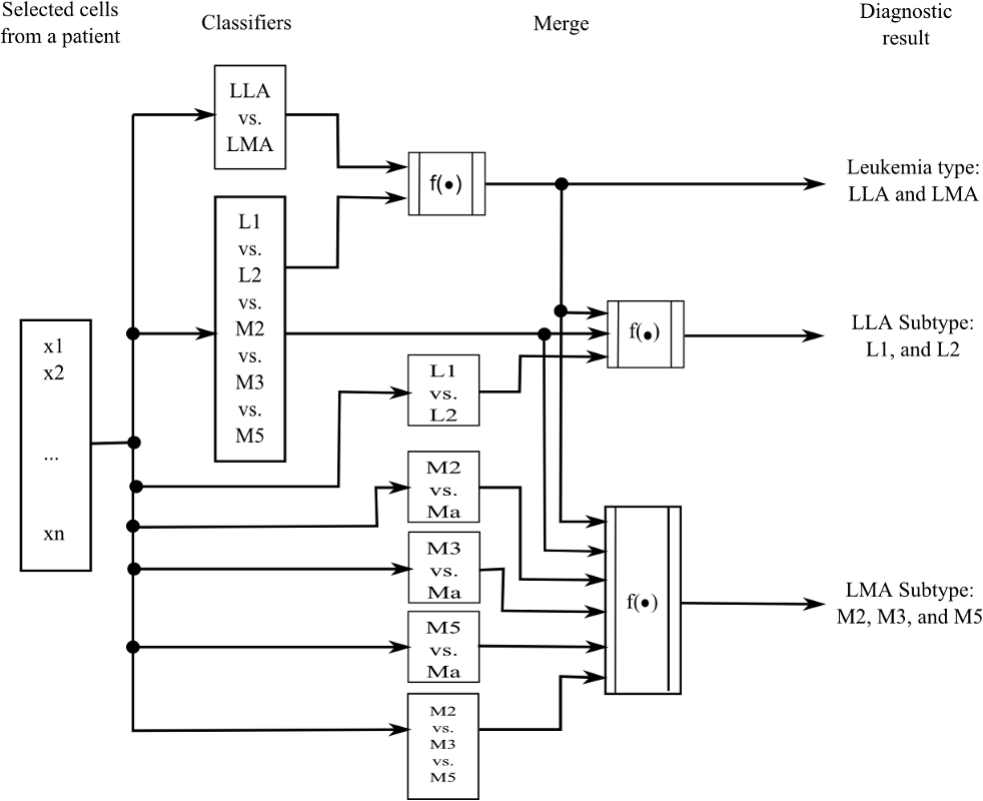
Classifiers fusion for the family and subtypes diagnosis of acute leukemia.

To lessen this problem, this work combines different classifiers to make the prediction of the corresponding acute leukemia family and subtypes. Fig 13 shows the scheme of the classifiers that were combined to determine the automatic diagnosis of acute leukemia in a patient.

As we can see in Fig 13, the proposed scheme considers information from the available patient cell samples and analyzes the class assignment results of different classifiers in order to take a decision.

The decision algorithm determines the patient's acute leukemia diagnosis using the majority vote technique over the classification of the samples cells.

**Fig 14:**
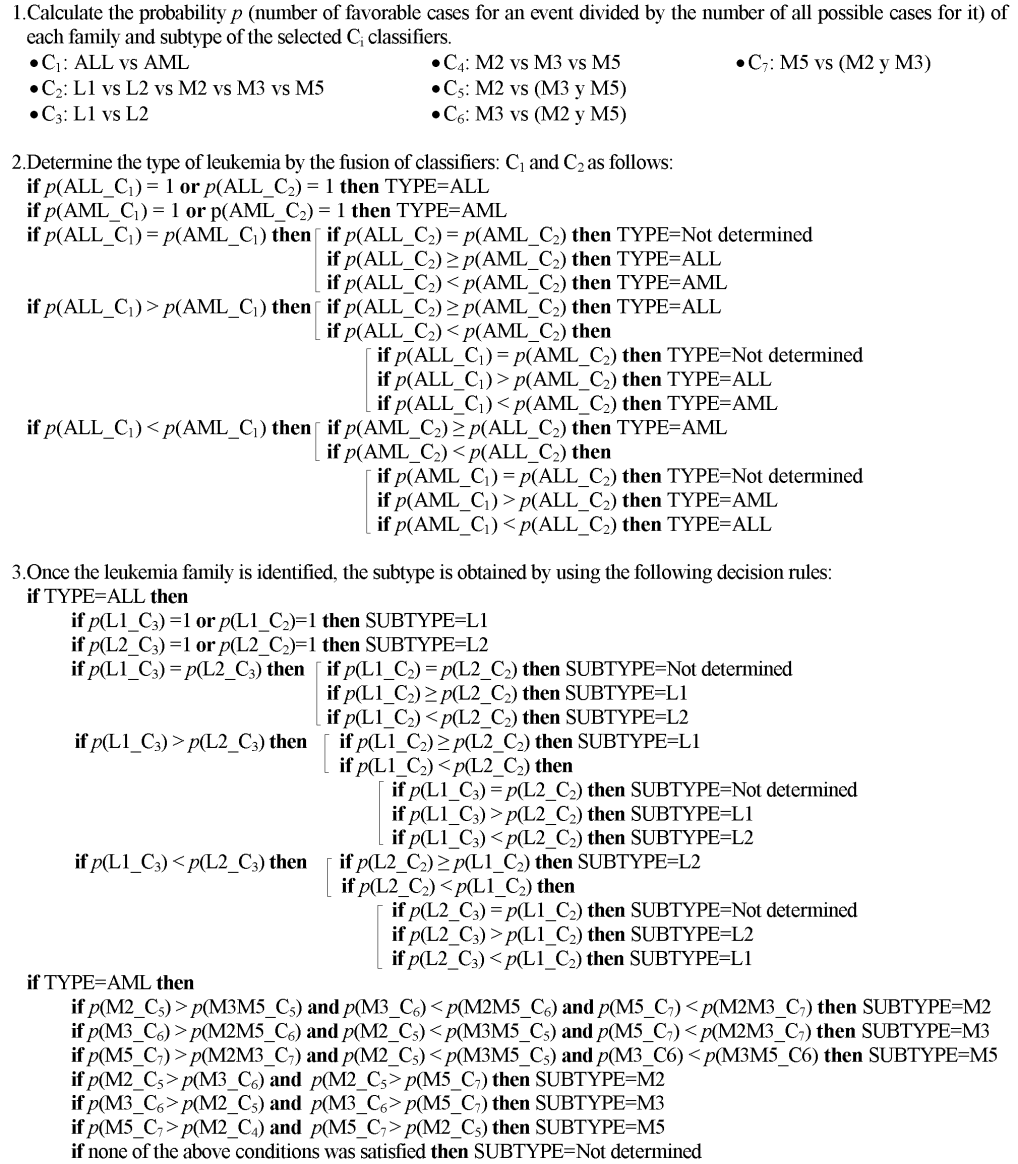
Diagnosis algorithm of types and subtypes of leukemia.

Fig 14 shows the steps for the proposed diagnosis algorithm to assign the acute leukemia family and subtypes of the input samples.

## Results

### Dataset description

In this work we used the Mexican Social Security Institute cells image collection that contains 633 bone marrow leukemia cells images with different color staining. These images were digitalized by [6] using a digital camera connected to a Carl Zeiss optical microscope with a 100x objective. Hence, all images have the same resolution. Tables 2 and 3 show the number of patients and samples of each family and subtype of acute leukemia included in the collection, respectively.

**Table 2:** Samples by subtypes.

Family	Subtype	No. Samples
**ALL**		**295**
	L1	102
	L2	135
	No subtype	58
**AML**		**338**
	M2	95
	M3	47
	M5	56
	No subtype	140

**Table 3:** Patients by subtypes.

Family	Subtype	No. Samples
**ALL**		**34**
	L1	14
	L2	15
	No subtype	5
**AML**		**29**
	M2	6
	M3	3
	M5	5
	No subtype	15

### Cell segmentation results

The proposed method showed good qualitative segmentation results allowing the extraction of the 633 leukocytes and their respective nucleus and cytoplasm. In order to measure the accuracy of the segmentation algorithm in a quantitative way, we tested it on a subset of the original images collection, which contains 20 leukemia cells images with a size of 256 x 256 pixels. It is important to notice that this test set includes different samples of images with color variations in their staining; furthermore, there are leukocytes overlapping with other blood cells.

Table 4 shows the evaluation results of the algorithm. This evaluation was obtained by comparing our automatic cells segmentation algorithm with cells manually segmented by an expert. The metrics used for evaluating the segmentation were: Precision = TP / P, FP Rate = FP / P, and FN Rate = FN / N, where TP and FP are the number of pixels correctly and incorrectly classified as cellular elements respectively, P is the number of pixels classified as cellular elements, FN is the number of pixels incorrectly classified as background, and N is the number of pixels classified as background.

**Table 4:** Evaluation of the cells segmentation algorithm.

	Precision	FP Rate	FN Rate
**nucleus**	95.87%	4.13%	2.33%
**cell**	95.75%	3.16%	3.83%

**Fig 15:**
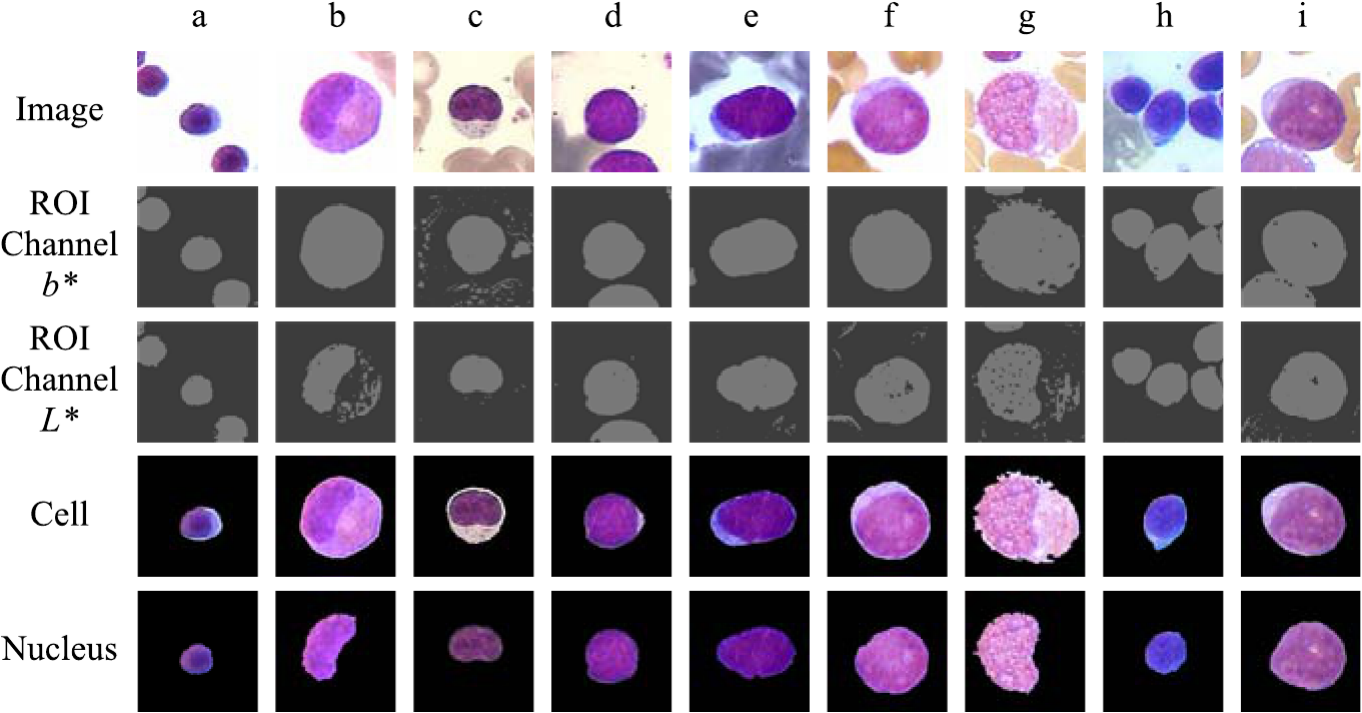
Examples of images segmentation with different staining and cell population.

Experimental results show that the proposed methodology allows the extraction of leukocytes and their respective nucleus from cells with good accuracy when experimenting with real bone marrow leukemia cells images. Fig 15 shows some examples with the results of the cell segmentation and identification.

As we can see in Fig 15, the leukemia cell images that we want to classify show different colors and textures. We can also see that leukocytes are overlapped with other blood cells. The algorithms previously used to segment cells images did not consider these conditions. Then, those algorithms were not able to work with images having variations in color staining, and high-cell population. This is the reason why we did not compare our results with previous works.

### Cell classification results

Experiments were performed by analyzing the nucleus, cell, and cytoplasm features as we propose in this work, and also by using only features taken from the whole cells as proposed in [4].

In each experiment we established different classification tasks to distinguish between families of acute leukemia (ALL and AML) subtypes of ALL (L1 and L2), and subtypes of AML (M2, M3, and M5). In the case of AML subtypes, we analyzed the behavior of a subtype with respect to the others performing binary and multiclass classifications. For each classification problem, we performed experiments using different types of features such as geometric, statistical, texture, size ratio, and eigen values. The classification was carried out using instance based classifiers, decision trees, regression functions as well as metaclassifiers available in [24]. Some of these classifiers are: k-Nearest Neighbor (IBk), Random Forest (RF), Simple Logistic (SL), Support Vector Machines (SMO), and Random Committee (RC), which were chosen because they were able to obtain the best results for acute leukemia subtypes classification in the work of [4].

The evaluation of the classification models was done using the 10 Fold cross-validation technique. The criteria for evaluating the classifiers were: overall percentage of correct classifications, true positive rate (TPR), true negative rate (TNR), and area under the ROC curve. Table 5 shows the best classifier for each experiment. These results were achieved using geometric, color, texture, and size ratio features as attributes. The eigen values did not represent useful information to distinguish among leukemia subtypes.

**Table 5:** Results of the best classifier for each type or subtype of acute leukemia cells.

Classification Problem	Classifier	Features*	Prec. %	TPR	TNR	AUC
**ALLvsAML**	SMO	N&C	92.20	0.920	0.924	0.921
**ALLvsAML**	SL	C	81.32	0.822	0.803	0.899
**L1vsL2**	IBk	N&C	84.40	0.835	0.853	0.907
**L1vsL2**	SL	C	76.78	0.667	0.845	0.814
**M2vs(M3&M5)**	RC.RF	N&C	92.45	0.883	0.962	0.959
**M2vs(M3&M5)**	RC.RF	C	74.24	0.706	0.778	0.805
**M3vs(M2&M5)**	IBk	N&C	91.89	0.805	0.955	0.880
**M3vs(M2&M5)**	RC.RF	C	80.79	0.390	0.940	0.788
**M5vs(M2&M3)**	IBk	N&C	91.89	0.870	0.938	0.955
**M5vs(M2&M3)**	RF	C	84.37	0.730	0.890	0.866
**M2vsM3vsM5**	RC.RF	N&C	88.39	0.904	0.894	0.945
**M2vsM3vsM5**	RC.RF	C	66.63	0.801	0.612	0.784

*For the Features column N stands for Nucleus and C for Cytoplasm.

Comparing the results obtained in the classification of leukemia cells using nucleus and cytoplasm features with the results obtained using only cell features, it can be clearly seen that the cell is better described by using features from its cellular elements.

### Diagnosis algorithm results

Tables 6 and 7 show the results of the algorithms that combine different binary and multiclass classifiers, respectively, for the diagnosis of the family and subtypes of acute leukemia from the smears samples available for a patient.

**Table 6:** Evaluation of the acute leukemia diagnosis algorithm by merging binary classifiers results.

Classification	No. examples	Correct	Not determined	Failed
**Types**	**63**	**92.0635%**	**4.7619%**	**3.1746%**
ALL	34	88.2352%	5.8824%	5.8824%
AML	29	96.5517%	3.4483%	0%
**Subtypes**	**43**	**81.3954%**	**11.6279%**	**6.9767%**
L1	15	80%	13.3333%	6.6667%
L2	14	78.5714%	14.2857%	7.1429%
M2	6	83.3333%	16.6667%	0%
M3	3	66.6667%	33.3333%	0%
M5	5	100%	0%	0%

**Table 7:** Evaluation of the acute leukemia diagnosis algorithm by merging multi-class classifiers results.

Classification	No. examples	Correct	Not determined	Failed
**Types**	**63**	**95.2381%**	**3.1746%**	**1.5873%**
ALL	34	94.1176%	2.9412%	2.9412%
AML	29	96.5517%	3.4483%	0.0000%
**Subtypes**	**43**	**90.6977%**	**4.6512%**	**4.6512%**
L1	15	86.6667%	6.6667%	6.6667%
L2	14	85.7143%	7.1429%	7.1429%
M2	6	100%	0%	0%
M3	3	100%	0%	0%
M5	5	100%	0%	0%

The diagnosis algorithm evaluation shows that by matching the information of different classifiers for all the patient's cells we were able to determine the family and subtype of acute leukemia with better accuracy. However, when we only use a few cells samples of a patient, we do not recommend using the diagnosis algorithm, since it is very sensitive to errors due to incorrect cells classification.

## Discussion

In this paper we proposed a novel method that uses color and texture information of images pixels in order to segment leukocytes and their respective nucleus and cytoplasm from bone marrow leukemia cells images.

We used the CIE L*a*b* color space and the 2D-Wold’s decomposition texture model as they allow us to analyze blood cells images in a similar way to the human visual perception system. We modeled the color and texture information by using a Markov Random Field in order to obtain regions of cell elements. We then extracted color, shape, and containment proportion information, and we used a cell identification algorithm to recognize cells and their respective nucleus and cytoplasm as well as overlapped regions. When there were cells or nucleus overlapping other cell elements, we separated them by using a cell separation algorithm based on linear interpolation in the polar space to provide a conical shape.

It is important to note that our method can be applied to images that show heterogeneous color and texture staining and high-cell population, a desirable property when working with bone marrow smears. Experimental results show that our method (with this type of images) achieves a segmentation accuracy of 95% (average) when it is compared with a manual segmentation performed by an expert.

With respect to the cell classification process, we can demonstrate that the use of descriptive features of the nucleus and cytoplasm of the cells improved their representation, allowing the classification of acute leukemia types and subtypes to significantly increase its accuracy (from 7% to 22% increase) compared with that obtained when we only use descriptive features of the whole cell.

With regard to the diagnosis algorithm, we found that by fusing different classifiers it was possible to reduce the number of false positives and false negatives that were present in different classifiers. This allowed us achieving an accuracy of 95% in the diagnosis of acute leukemia families and an accuracy of 90% in subtypes diagnosis.

The above results showed that the use of contextual information in the cells segmentation, identification, and classification process can be efficiently applied in the diagnosis of families and subtypes of acute leukemia.
