# Correction: Segmentation and Classification of Bone Marrow Cells Images Using Contextual Information for Medical Diagnosis of Acute Leukemias

**DOI:** 10.1371/journal.pone.0134066

**Published:** 2015-07-24

**Authors:** Carolina Reta, Leopoldo Altamirano, Jesus A. Gonzalez, Raquel Diaz-Hernandez, Hayde Peregrina, Ivan Olmos, Jose E. Alonso, Ruben Lobato

The affiliation for the sixth author is incorrectly listed in English. The correct affiliation is: Faculty of Computer Science, Benemérita Universidad Autónoma de Puebla, Puebla, Mexico.
